# Machine learning progressive CKD risk prediction model is associated with CKD-mineral bone disorder

**DOI:** 10.1016/j.bonr.2024.101787

**Published:** 2024-07-04

**Authors:** Joseph Aoki, Omar Khalid, Cihan Kaya, Tarush Kothari, Mark Silberman, Con Skordis, Jonathan Hughes, Jerry Hussong, Mohamed E. Salama

**Affiliations:** Sonic Healthcare USA, 12357A - A Riata Trace Parkway, Suite 210, Austin, TX 78727, USA

**Keywords:** Chronic kidney disease, Chronic kidney disease-metabolic bone disorder, Machine learning, Parathyroid hormone, Calcium, Phosphate

## Abstract

**Background:**

Recently, we developed the machine learning (ML)-based Progressive CKD Risk Classifier (PCRC), which accurately predicts CKD progression within 5 years. While its performance is robust, it is unknown whether PCRC categorization is associated with CKD-mineral bone disorder (CKD-MBD), a critical, yet under-recognized, downstream consequence. Therefore, we aimed to 1) survey real-world testing utilization data for CKD-MBD and 2) evaluate ML-based PCRC categorization with CKD-MBD.

**Methods:**

The cohort study utilized deidentified data from a US laboratory outpatient network, composed of 330,238 outpatients, over 5 years. The main outcomes were: 1) Laboratory testing utilization of eGFR, urine albumin creatinine ratio (UACR), parathyroid hormone (PTH), calcium, phosphate; and 2) PCRC categorization and biochemical abnormalities associated with CKD-MBD over 5 years.

**Results:**

We identified significant under-utilization of laboratory testing for UACR, phosphate and PTH, which ranged from −40 % to −100 % against the minimum standard-of-care. At five years, the CKD progression group, as predicted by the PCRC, was associated with 15.5 % increase in phosphate (*P* value <<0.01) and 94.9 % increase in PTH (P value <<0.01), consistent with CKD-MBD.

**Conclusions:**

We identified significant under-utilization of laboratory testing for CKD-MBD. Moreover, we demonstrated that CKD progression, as predicted by the PCRC, is associated with CKD-MBD, several years in advance of disease. To our knowledge, this investigation is the first to examine the role of predictive analytics for CKD progression on mineral bone disorder. While further studies are required, these findings have the potential to advance AI/ML-based risk stratification and treatment of CKD and CKD-MBD.

## Highlights

• Testing for chronic kidney disease-mineral bone disorder is significantly under-utilized
• Machine learning model is associated with abnormalities in parathyroid hormone
• Machine learning improves risk stratification for chronic kidney disease-mineral bone disorder

## Graphical abstract

**Figure:**
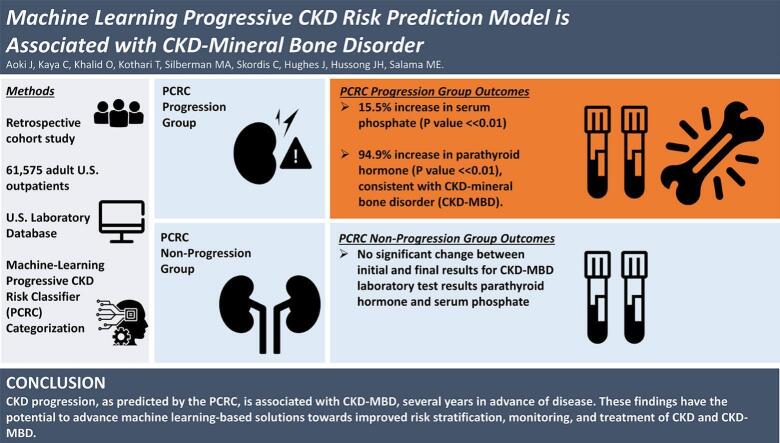

## Abbreviations

CKD: chronic kidney disease
ML: machine learning
UACR: urine albumin creatinine ratio
PCRC: Progressive CKD Risk Classifier
MBD: mineral bone disorder
KDIGO: Kidney Disease Improving Global Outcomes
PTH: parathyroid hormone

## 1 Introduction

Chronic kidney disease (CKD) progression is characterized by accelerated decline in eGFR, leading to multi-system dysfunction, increased healthcare expenditure, and poor clinical outcomes (Stevens et al., 2013; Hemmelgarn et al., 2009; Golestaneh et al., 2017). Among the most significant sequelae of CKD progression, mineral bone disorder (MBD) affects nearly all of the 37 million Americans with CKD and is a tremendous source of morbidity, healthcare costs, and mortality (Centers for Disease Control and Prevention, 2023; *Health*, 2021; Schumock et al., 2008; Magagnoli et al., 2023).

Kidney Disease Improving Global Outcomes (KDIGO) Clinical Practice Guidelines define CKD-MBD as a systemic disorder with abnormalities involving mineral metabolism, bone, and/or vascular/soft-tissue calcification (Wang et al., 2017). Identified as early as CKD stage G3a, patients with CKD-MBD demonstrate serum changes in numerous minerals and hormones, including: elevated parathyroid hormone (PTH), decreased calcium, decreased vitamin D, and/or increased phosphate (Wang et al., 2017). These biochemical abnormalities are the primary drivers in bone and calcium pathology, culminating in substantially increased risk of fractures, cardiovascular disease, and death (Chue et al., 2011; Bhan et al., 2010; Martin and González, 2007; Floege et al., 2011; Palmer et al., 2011). Despite its grave consequences, studies suggest biochemical evaluation for CKD-MBD represents a major care gap among academic and/or large healthcare networks (Wetmore et al., 2021). However, the utilization CKD-MBD laboratory testing among the general US outpatient population is not fully characterized.

Owing to its high biological and clinical complexity, artificial intelligence (AI)/machine learning (ML) has the potential to address care deficiencies associated with CKD-MBD (Lederer et al., 2023; Gaweda et al., 2022). Foundational work by Peterson and Riggs generated a physiologically-based mathematical model of calcium homeostasis and bone biology (Peterson and Riggs, 2010). Their novel systems-based apporach incorporated varied biological data (e.g., calcium, PTH, phosphate and other biomarkers) to create an in silico construct that has formed the basis for advances in diagnosis and therapeutics across a wide spectrum of bone disease (Peterson and Riggs, 2015; Berkhout et al., 2015; Rodriguez et al., 2016). Leveraging machine learning, Rodgriguez et al. extracted data from a cohort of 1758 patients underoing hemodialysis and demonstrated unique interactions between mineral metabolism parameters (Rodriguez et al., 2016). Together, these investigations illustrate the tremendous progress and unique opportunity of AI/ML to improve healthcare across major bone diseases, including CKD-MBD.

Recently, we developed the ML Progressive CKD Risk Classifier (PCRC) in order to advance timely, evidence-based interventions and prevent adverse events (Aoki et al., 2023). Trained and validated on a large US laboratory information system cohort, composed of 110,264 patients using machine learning methods, the 7-variable risk classifier uses routine laboratory parameters (age, sex, eGFR, eGFR slope, UACR, serum albumin, serum albumin slope) to accurately predict CKD progression within 5 years. While the performance of the Progressive CKD Risk Classifier is accurate (AUC = 0.85), it is unknown whether patients predicted to develop progressive CKD by the machine learning PCRC are also associated with clinically significant, downstream complications such as CKD-MBD (Aoki et al., 2023).

Against this background, we aimed to leverage longitudinal real-world laboratory data to: 1) broadly survey CKD-MBD testing utilization across a diverse, general US outpatient cohort and, 2) examine the association between Progressive CKD Risk Classification and biochemical abnormalities associated with CKD-MBD.

## 2 Methods

### 2.1 Study population and setting

The initial cohort was identical to the study population, as previously described (Aoki et al., 2023). Briefly, the population included de-identified laboratory information system (LIS) data from 330,238 outpatient participants across geographically diverse (i.e., Northeast, Southwest, Mid-South, and West/Pacific) regions of the U.S. The cohort selected for patients with dates of service between January 1, 2017 and December 31, 2021. The minimum criteria for inclusion in the study also included two eGFR values over the span of at least 48 months (i.e., one eGFR value in 2017 and one in 2021). At the outset, these inclusion criteria ensured optimal follow-up with no missingness in data over the specified time. Sub-group analysis cohorts for CKD-MBD required at least two measurements of serum calcium, PTH, and/or phosphate within 5 years. Ethical approval was waived by Western Copernicus Group Institutional Review Board in view of the retrospective nature of the study and all the procedures being performed were part of routine care.

### 2.2 Data collection & measurements

Biochemical testing for creatinine/eGFR, UACR, calcium, parathyroid hormone, and phosphate were performed on equivalent high-throughput instrument platforms across all testing sites. Creatinine testing was standardized according to best practices, including calibration traceable to an isotope dilution mass spectrometry (IDMS) reference measurement procedure. eGFR was calculated using the 2009 CKD Epidemiology Collaboration (CKD-EPI) creatinine equation without the race-based coefficient (Levey et al., 2009). Whereas, the vast majority of creatinine/eGFR, calcium testing were ordered as part of the highly utilized basic metabolic panel (BMP) and comprehensive metabolic panel (CMP), testing for phosphate and parathyroid hormone testing was performed almost entirely as standalone orders.

Established according to national society and/or manufacturer guidelines, reference ranges for calcium, parathyroid hormone, and phosphate were 8.5–10.5 mg/dL, 15–65 pg/ml, and 2.5–4.5 mg/dL, respectively (CLSI, 2014). By extension, biochemical evidence of CKD-MBD was defined as values outside the aforementioned reference ranges (American Society for Clinical Pathology, 2018).

CKD staging was determined according to the 2012 KDIGO Clinical Practice Guidelines based on estimated Glomerular Filtration Rate (≥90 [G1], 60–89 [G2], 45–59 [G3a], 30–44 [G3b], 15–29 [G4], <15 ml/min/1.72m^2^ [G5]) and urine albumin creatinine ratio (UACR) (<30 [A1], 30–299 [A2], ≥300 mg/g creatinine [A3]) (Stevens et al., 2013).

CKD and CKD-MBD testing frequency was compared against minimum international guideline recommendations for select analytes: G1 = 0–1 eGFR tests per year, 0–1 UACR test per year; G2 = 0–1 eGFR tests per year, 0–1 UACR test per year; G3a = 1–3 eGFR tests per year, 1–3 UACR test per year, 1–2 calcium and phosphate tests per year, 0–1 PTH tests per year; G3b = 2–3 eGFR tests per year, 2–3 UACR test per year, 1–2 calcium and phosphate tests per year, 0–1 PTH tests per year; G4 = 3–4+ eGFR tests per year, 3–4+ UACR test per year, 2–4 calcium and phosphate tests per year, 1–2 PTH tests per year; G5 = 4+ eGFR tests per year, 4+ UACR test per year, 4–12 calcium and phosphate tests per year, 2–4 PTH tests per year (Stevens et al., 2013; Wang et al., 2017).

As previously described (Aoki et al., 2023), the machine learning Progressive CKD Risk Classifier model was built on a large US laboratory information system cohort. Training and testing datasets were generated, using 80 % of the data in training and 20 % for independent testing, respectively. After filtering, a random forest (RF) classifier was constructed using sklearn version 1.1.1. Ten thousand trees were generated within the models to develop the classifier. Additional 5-fold cross validation was performed on the training set, and a representative model was used in the testing set to assess the performance. R version 4.2.1 was used to perform additional data wrangling and statistical analysis using the tidyverse packages and the equivalence package for statistical testing. In time-to-event analysis, participants were censored if the event (i.e., >30 % eGFR decline) occurred during the designated slope evaluation period. In addition, calibration was performed with additional time series data going from 6 months to 36 months, and the 95 % confidence interval was generated for the model. During development of the PCRC model (Aoki et al., 2023), the following parameters were compared, normalized, and/or optimized: isotonic versus sigmoid model calibration; linear versus log scale; 6-month versus 12-month time intervals. The overall predictive accuracy was assessed using the area under the receiver-operator characteristic curve (AUC) on the test set.

For the current study, the machine learning Progressive CKD Risk Classifier was selected for the following reasons: 1) The PCRC is accurate with an AUC of 0.85; 2) the PCRC demonstrates broad usability because it utilizes existing and readily obtainable laboratory information (American Society for Clinical Pathology, 2018); 3) the PCRC is uniquely based on ML random forest analysis, which incorporates continuous and/or longitudinal features and may be less prone to variance when compared to logistic regression and other methods (Miao et al., 2015; Zhou et al., 2016; Wang and Li, 2017). Due to extensive training, testing, and optimization during development, additional parameter tuning or modification of the PCRC model was not performed for this study. By retaining the original model specifications, sources of bias and error were also limited.

### 2.3 Metrics and statistical analysis

For CKD and CKD-MBD laboratory testing utilization, descriptive statistics (e.g., testing frequency, means, interquartile ranges, and percentages) were generated according to initial KDIGO CKD stage and compared against international guideline recommendations (Stevens et al., 2013; Chue et al., 2011). To evaluate for an association with CKD-MBD, the Progressive CKD Risk Classifier categorized participants into either: CKD progression (i.e., ≥ 30 % decline in eGFR predicted within 5 years) versus non-progression (i.e., <30 % eGFR decline predicted within 5 years) groups. Biochemical testing data for kidney function (eGFR) and/or CKD-MBD (PTH, calcium, and phosphate) were then compared between initial and final results according to PCRC category using paired *t-*test. F1 score and recall were also used to assess the performance of the model. Due to the lack of access to laboratory results for key metabolites (e.g. 1,25(OH)_2_D and 25(OH)D)) within the dataset, comprehensive evaluation for whether PCRC categorization is associated with a change in vitamin D levels over time was not evaluated in this study.

## 3 Results

### 3.1 Baseline characteristics and utilization rate for CKD and CKD-MBD testing

The initial registry included 330,238 adult patients ages 18 to 75 years old with an outpatient clinical laboratory encounter within the Sonic Healthcare USA network between January 1, 2017 and December 31, 2021. In order to control for the well-established challenge of interlaboratory variation, all biochemical analyses were performed on equivalent high-throughput instrument platforms and standardized according to best practices, including calibration traceable to an isotope dilution mass spectrometry (IDMS) reference measurement procedure (Myers et al., 2006; Alfego et al., 2021; Kausz et al., 2005).

**Fig. 1:**
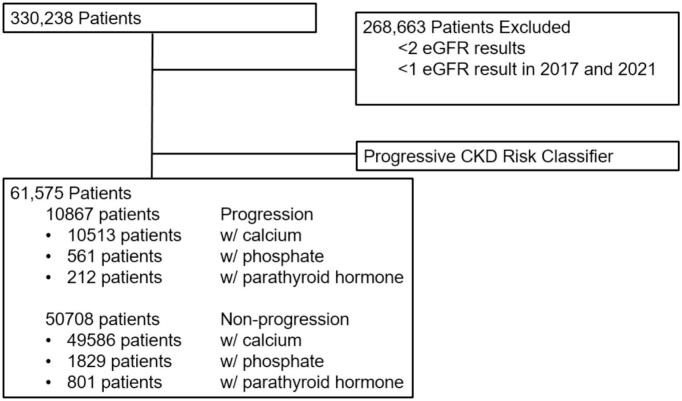
Participant flow diagram. The flow diagram depicts the number of adult participants between 18 and 75 years of age in the original data set prior to removal due to exclusion criteria. Omitted participants included those with less than two eGFR values at least 48 months apart. The final data set, composed of 61,575 participants, was then categorized by the Progressive CKD Risk Classifier as either Progression or Non-progression sub-groups.

After applying inclusion criteria and stratifying the participants according to eGFR stage, the cohort contained 61,575 patients with at least 48 months of follow up data (Fig. 1). The cohort included participants with a broad spectrum of baseline eGFR results associated with G1 (33%) G2 (42%), G3a (17 %), G3b (6.5 %), G4 (1.8 %), and G5 (0.5 %). The average age and sex distribution ranges across the GFR subgroup categories were 55 to 65 years-old and 45 % to 54 % male, respectively. While the overall follow-up and eGFR testing utilization was adequate (up to three eGFR tests per year), the frequency of essential kidney function (i.e., UACR) and CKD-MBD (i.e., PTH, and phosphate) testing was significantly less than one result per year. To be sure, the median UACR testing frequency in our cohort for G3-G4 disease was less than one (0.4) time per year, which represents a deficiency of −60 % to −80 % against the minimum standard-of-care. For metabolic bone function (phosphate and PTH) testing, the utilization deficiency was −50 % to −90 % and − 40 % to −100 % of international guideline recommendations, respectively (Table 1).

**Table 1:** Baseline Characteristics and CKD/CKD-MBD Laboratory Testing Utilization over 5 years. GFR, glomerular filtration rate. UACR/yr, urine albumin creatine ratio tests per year. Calc/yr, serum calcium tests per year. Phos/yr, serum phosphate tests per year. PTH/yr, parathyroid hormone tests per year. IQR, interquartile range. Δ SOC, percent change compared with the minimum standard-of-care as described in KDIGO Clinical Practice Guidelines.

GFR category	GFR category (%)	Age (IQR)	Male (%)	eGFR/yr (IQR)	Δ SOC, eGFR	UACR/yr (IQR)	Δ SOC, UACR	Calc/yr (IQR)	Δ SOC, Calc	Phos/yr (IQR)	Δ SOC, Phos	PTH/yr (IQR)	Δ SOC, PTH
G1	20,320 (33)	55 (15)	10,160 (50)	1.6 (2.2)	NA	0.6 (0.6)	NA	1.6 (1.2)	NA	0 (0)	NA	0 (0)	NA
G2	25,862 (42)	63 (11)	13,448 (52)	1.8 (1.2)	NA	0.4 (0.6)	NA	1.8 (1.2)	NA	0 (0)	NA	0 (0)	NA
G3a	10,468 (17)	64 (9)	5234 (50)	2.2 (1.6)	120 %	0.4 (0.6)	−60 %	2.2 (1.6)	120 %	0.4 (0.4)	−60 %	0 (0.2)	NA
G3b	4002 (6.5)	65 (9)	1801 (45)	2.6 (1.8)	160 %	0.4 (0.6)	−80 %	2.4 (1.8)	140 %	0.4 (1.2)	−60 %	0.2 (0.8)	NA
G4	1108 (1.8)	63 (11)	1108 (48)	3 (2.2)	0 %	0.4 (0.6)	−87 %	2.8 (2.2)	40 %	1 (2)	−50 %	0.6 (1.4)	−40 %
G5	308 (0.5)	59 (15)	155 (54)	2.2 (2.2)	−45 %	0.2 (0.4)	−95 %	2 (2)	−50 %	0.4 (1.6)	−90 %	0 (0.8)	−100 %

### 3.2 CKD progression risk classification and CKD-MBD biochemical abnormalities

To evaluate a potential association with CKD-MBD, the machine learning Progressive CKD Risk Classifier leveraged routine laboratory values and categorized the cohort as either progression (i.e., eGFR predicted to decline by ≥ 30 % within 5 years) or non-progression (i.e., eGFR predicted to decline by <30 % within 5 years). The CKD progression and non-progression groups were then compared against average initial and final results for eGFR, calcium, PTH, and phosphate. Overall, the performance of the classifier was robust, and the F1 score and recall score were 0.85 and 0.86, respectively. Follow up time between the first and last result for each parameter was also strong and ranged from 36.4 months (PTH) to 51.1 months (eGFR).

**Fig. 2:**
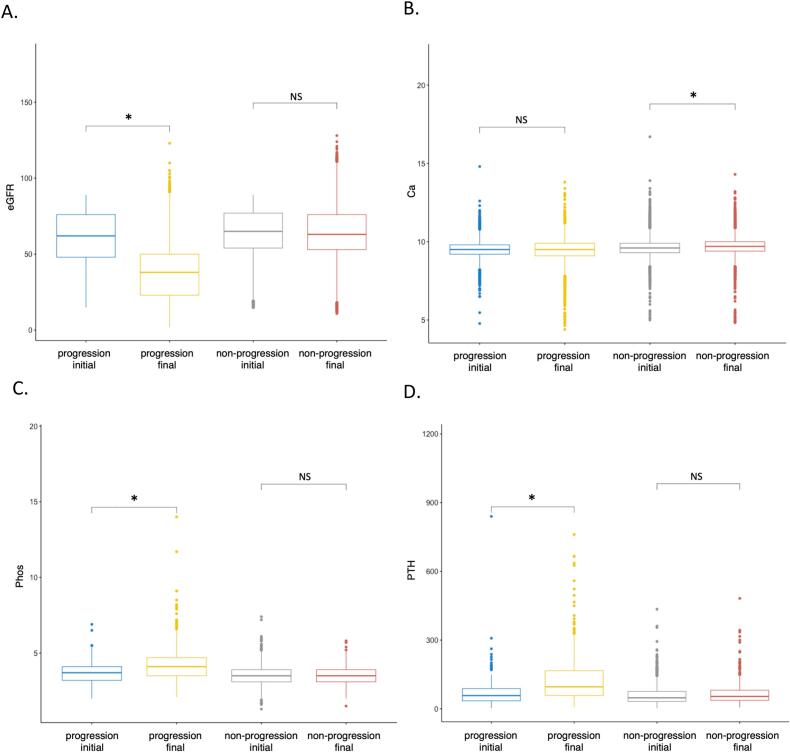
A-D. Progressive CKD Risk Classifier Categorization and CKD-MBD Biochemical Changes. A) eGFR, estimated glomerular filtration rate. B) Ca, serum calcium. C) Phos, serum phosphate. D) PTH, parathyroid hormone. *, *p* value <<0.001. NS, not-significant, p value >0.05.

As predicted, initial eGFR versus final eGFR declined by 38.6 % over 5 years in the CKD progression group (60.6 vs 37.2 ml/min/1.72m^2^, *P* value <<0.01). Unlike the non-progression group, the CKD progression group was also associated with 15.5 % increase in phosphate (3.68 vs 4.25 mg/dL, P value <<0.01) and 94.9 % abnormal increase in PTH (72.3 vs 140.9 pg/dL, P value <<0.01), compatible with CKD-MBD (Fig. 2. and Table 2).

**Table 2:** Progressive CKD Risk Classifier Categorization and CKD-MBD, Summary. CKD-MBD, chronic kidney disease mineral bone disorder. IQR, interquartile range. PTH, parathyroid hormone.

	CKD Progression				CKD Non-progression			
Analyte	Initial (IQR)	Final (IQR)	% change	*p*-value	Initial (IQR)	Final (IQR)	% change	p-value
eGFR, ml/min/1.72 m^2^	60.6 (28)	37.2 (27)	−38.6 %	<<0.01	64.1 (23)	63.9 (23)	−0.3 %	0.06
Calcium, mg/dl	9.48 (0.6)	9.47 (0.8)	−0.1 %	0.09	9.64 (0.6)	9.69 (0.6)	0.5 %	<<0.01
Phosphate, mg/dL	3.68 (0.9)	4.25 (1.2)	15.5 %	<<0.01	3.52 (0.8)	3.50 (0.8)	−0.6 %	0.07
PTH (pg/ml)	72.3 (53.3)	140.9 (108.3)	94.9 %	<<0.01	61.9 (44)	66.4 (44)	7.3 %	0.05

## 4 Discussion

Leveraging longitudinal laboratory data across a diverse US outpatient population, this retrospective cohort study demonstrated substantial under-utilization of laboratory testing for kidney function and CKD-MBD evaluation. In agreement with prior studies (Bhan et al., 2010; Wetmore et al., 2021; Alfego et al., 2021; Kausz et al., 2005), we identified significant deficiency in UACR (−60 % to −80 %), PTH (−40 % to −100 %) and phosphate (−50 % to −90 %) testing compared against the minimum standard-of-care (Stevens et al., 2013; Wang et al., 2017). Particularly for CKD-MBD, the present findings represent the largest reported survey for CKD-MBD testing in a general (i.e., non-academic, non-large healthcare network) US outpatient population. Together, the data are troubling because assessment of these parameters is essential for accurate diagnosis and management.

Possible reasons for inadequate laboratory testing for CKD and CKD-MBD are myriad. Firstly, up to 90 % of patients with CKD (including 40 % of patients with G4/G5 disease) are unaware of their diagnosis, which preclude timely clinical-laboratory evaluation of CKD and its sequelae, such as mineral bone disorder (Chu et al., 2020; Wang et al., 2022). Secondly, passive testing for some kidney function analytes (i.e., creatinine/eGFR, calcium) nearly always occurs as part of clinical orders built within routine, highly-utilized metabolic panels (e.g., complete metabolic or basic metabolic panels) (Alfego et al., 2021). However, testing for other critical CKD and/or CKD-MBD analytes - UACR, parathyroid hormone, phosphate - are not part of the BMP or CMP and require a standalone test order. Along with the lack of awareness, this fixed composition may, in part, explain the severe under-utilization of laboratory testing as observed in this study. While these challenges are concerning, they also raise the possibility that the addition of analytes (e.g., phosphate and UACR) to established metabolic panels could provide a solution to advance the diagnosis and timely management of CKD and CKD-MBD.

We then examined the role of Progressive CKD Risk Classification on CKD-MBD; and, to our understanding, the results herein are the first to demonstrate that AI/ML-based analytics for accelerated eGFR decline is associated with an elevated risk for CKD-mineral bone disorder. Particularly in the CKD Progression group, we observed significant increase in PTH and decrease in phosphate, several years following PCRC categorization. There was no significant change in serum calcium levels over time. While the exact mechanism(s) has not been fully elucidated, the leading theory is that, especially in moderate to advanced stages of CKD (CKD stage 3–4), calcium homeostasis is maintained by rising PTH. As CKD progresses further to kidney failure, this compensatory pathway cannot be sustained, and hypocalcemia ensues (Martin and González, 2007). This phenomenon may partially explain why patients with progressive CKD seen in this study demonstrated elevated PTH without hypocalcemia.

These findings have several applications. Firstly, they support the overall effectiveness and performance of the machine learning PCRC in predicting CKD progression and clinically significant, downstream sequelae. Secondly, the results provide evidence to support employing AI/ML-based analytics to stratify patients according to PCRC category, then, potentially, deploying targeted diagnostic (e.g., biochemical screening and monitoring), preventive (e.g., phosphate intake reduction), and/or therapeutic measures to raise awareness, restore bone-mineral homeostasis, and, ultimately, address CKD-MBD (Kausz et al., 2005).

Recently, Li et al. (Li et al., 2024) and Gaweda et al. (Gaweda et al., 2021) successfully generated AI/ML models for CKD-MBD. Combined, their work represents a sizable component of this emerging field reported in the literature. While there are similarities, the current study uniquely centers on leveraging the machine learning Progressive CKD Risk Classifier to identify which CKD patients have the highest risk of developing CKD-MBD, several years in advance of disease. By contrast, the outcome of interest for the Li and Gaweda models were focused primarily on other components of CKD-MBD care: biomarker discovery and therapeutic management, respectively (Li et al., 2024; Gaweda et al., 2021). Together, these data underscore the overall success of and potential for AI/ML on various facets of CKD-MBD. Indeed, the findings from the evolving field are dynamic and prospective studies are required to establish the optimal AI/ML model(s) for advancing the risk prediction, diagnosis, and therapy for CKD-MBD.

This study has several strengths. The cohort was derived from a general US outpatient population with up to five years of follow-up data, curated to ensure minimal missingness. Moreover, the biochemical data - some of which are known to demonstrate considerable inter-laboratory variability - were procured from equivalent high-throughput instrument platforms and validated according to best practices (CLSI, 2014; Myers et al., 2006; Souberbielle et al., 2006). Together, these features support the overall quality of the real-world data and generalizability of the findings.

There are, however, limitations to this investigation. To limit patients lost to follow-up, the cohort was restricted to participants with at least two eGFR measurements over five years, which may select for persons with higher health awareness. Compared with the findings seen here, the contrast between the current participants and an unselected outpatient population, widely sampled for CKD-MBD, may be starker. Additionally, the study did not interrogate for notable risk factors for CKD-MBD, including body mass index (Kausz et al., 2005), bone density (Iseri et al., 2020; Pazianas and Miller, 2021), other biomarkers (e.g., FGF-23) (Isakova et al., 2011), bone pathology (Fusaro et al., 2022), and nutrition history/medications (Rysz et al., 2021; Sultana et al., 2015). These shortcomings and others should be addressed in prospective randomized controlled trials.

In conclusion, this retrospective cohort study achieved its dual objectives by: 1) brightening the spotlight on significant under-utilization of kidney function and CKD-MBD laboratory testing; and 2) demonstrating that Progressive CKD Risk Classification is associated with CKD-mineral bone disorder. To our knowledge, this original investigation is the first to report an association between AI/ML-based predictive analytics for CKD progression and CKD-mineral bone disorder. While further, prospective studies are warranted, these findings have the potential to advance AI/ML solutions to improve risk stratification, diagnosis, and treatment of CKD and CKD-MBD.

## Funding

This research did not receive any specific grant from funding agencies in the public, commercial, or not-for-profit sectors.

## Ethical approval

This research received ethical approval from Western Copernicus Group Institutional Review Board.

## Informed consent

Informed consent was waived by Western Copernicus Group Institutional Review Board in view of the retrospective nature of the study and all the procedures being performed were part of routine care.

## CRediT authorship contribution statement

**Joseph Aoki:** Writing – original draft, Investigation, Formal analysis, Conceptualization. **Omar Khalid:** Writing – review & editing, Formal analysis, Data curation, Conceptualization. **Cihan Kaya:** Writing – review & editing, Formal analysis, Data curation, Conceptualization. **Tarush Kothari:** Writing – review & editing. **Mark Silberman:** Writing – review & editing. **Con Skordis:** Writing – review & editing, Data curation. **Jonathan Hughes:** Writing – review & editing. **Jerry Hussong:** Writing – review & editing, Supervision. **Mohamed E. Salama:** Writing – review & editing, Supervision, Conceptualization.

## Declaration of competing interest

The authors declare they have no conflicts of interest to disclose.

## Data Availability

Data will be made available on request.
